# A systematic overview of the literature regarding group prenatal care for high-risk pregnant women

**DOI:** 10.1186/s12884-017-1522-2

**Published:** 2017-09-29

**Authors:** Brittany M. Byerley, David M. Haas

**Affiliations:** Department of OB/GYN, Indiana University School of Medicine, 550 N. University Blvd, UH 2440, Indianapolis, IN 46202 USA

## Abstract

**Background:**

Group prenatal care (GPC) models have been gaining popularity in recent years. Studies of high-risk groups have shown improved outcomes. Our objective was to review and summarize outcomes for women in GPC for women with specific high-risk conditions.

**Methods:**

A systematic literature review of Ovid, PubMed, and Google Scholar was performed to identify studies reporting the effects of group prenatal care in high-risk populations. Studies were included if they reported on pregnancy outcome results for women using GPC. We also contacted providers known to be utilizing GPC for specific high-risk women. Descriptive results were compiled and summarized by high-risk population.

**Results:**

We identified 37 reports for inclusion (8 randomized trials, 23 nonrandomized studies, 6 reports of group outcomes without controls). Preterm birth was found to be decreased among low-income and African American women. Attendance at prenatal visits was shown to increase among women in GPC in the following groups: Opioid Addiction, Adolescents, and Low-Income. Improved weight trajectories and compliance with the IOM’s weight recommendations were found in adolescents. Increased rates of breastfeeding were found in adolescents and African Americans. Increased satisfaction with care was found in adolescents and African Americans. Pregnancy knowledge was increased among adolescents, as was uptake of LARC. Improved psychological outcomes were found among adolescents and low-income women. Studies in women with diabetes demonstrated that fewer women required treatment with medication when exposed to GPC, and for those requiring treatment with insulin, GPC individuals required less than half the dose. Among women with tobacco use, those who had continued to smoke after finding out they were pregnant were 5 times more likely to quit later in pregnancy if they were engaged in GPC.

**Conclusions:**

Several groups of high-risk pregnant women may have benefits from engaging in group prenatal care. Because there is a paucity of high-quality, well-controlled studies, more trials in high-risk women are needed to determine whether it improves outcomes and costs of pregnancy-related care.

**Electronic supplementary material:**

The online version of this article (10.1186/s12884-017-1522-2) contains supplementary material, which is available to authorized users.

## Background

Prenatal care has been widely implemented as a means to improve health outcomes for both mothers and babies. Group prenatal care (GPC) is an increasingly popular system of delivering prenatal care. GPC allows women to come together as a support system while both receiving prenatal care and participating in education. There are currently many models of GPC, including CenteringPregnancy®, CenteringPregnancy Plus, and Expect With Me, to name a few. CenteringPregnancy®‘s model of GPC is comprised of three major components: health assessment, education, and support. Groups are composed of 8–12 women. Sessions generally last 90–120 min and the women meet along with their health provider and group facilitator about 10 times during their pregnancy. This interactive approach empowers women to take control of their health during their pregnancy. Many of these elements are common to different models of GPC.

While organizations such as CenteringPregnancy®‘s developer, Centering Health International, tout improved outcomes in women receiving GPC, studies report mixed results. Some studies have shown significant decreases in preterm birth rates [1–4], including a study by Ickovics showing a reduced risk by 30% overall or 41% in African American women [5]. However, other studies failed to show any difference in preterm birth rates [6–12]. The synthesized data meta-analysis of both randomized and observational studies found no difference in preterm birth rates with the exception of rates in African American women [13]. Rate of low birth weight infants appears to be significantly reduced in a recent meta-analysis [13]. Rates of cesarean section also trend down for women in GPC [1, 2, 7, 11, 12, 14]. Admission rates to the neonatal intensive care unit (NICU) are largely unaffected [9, 10, 12, 15]. Breastfeeding rates generally appear to be increased for women in GPC [1, 5, 8, 14, 16–18], including higher rates of both initiation and continuation. GPC has been consistent in demonstrating increased patient satisfaction with care over standard prenatal care [19], increased knowledge about pregnancy and labor [19, 20], and increased readiness for labor and delivery [20].

While there is still much to be learned regarding GPC in unselected populations, preliminary results offer the possibility of improved outcomes in high risk populations. The meta-analysis mentioned above showed an overall risk reduction of preterm birth of 3 per 100 live births in African American women [13], which may be of significance as the rate of preterm birth is much higher in this population: 13.23% compared to 8.91% for whites [21]. However, the meta-analysis did not explore other subgroups of high-risk pregnant women. The objective of this report is to collate and present results from using GPC models for women in specific high-risk categories. These groups include: women with diabetes, women with tobacco or opioid abuse, pregnant adolescents, African Americans, low-income and homeless women, overweight/obese women, and those infected with HIV/AIDS.

## Methods

We utilized two strategies to identify all relevant data concerning group prenatal care that specifically targeted high-risk pregnant women and groups. First, we performed a systematic literature review using the following search engines: Ovid, PubMed, and Google Scholar. All searches for databases were performed from inception through March 9, 2017. We used the search terms “Group Antenatal Care,” “Group Prenatal Care,” “CenteringPregnancy,” “Centering Pregnancy,” and “Expect with Me.” As a program reporting outcomes of Centering Pregnancy Plus would have been identified by “Centering Pregnancy,” we did not include the word “plus” in the search. Mothers 2 Mothers is a program of prenatal care based on a mentorship model that encompasses some elements of GPC. As the Mothers2Mothers program was more about 1-on-1 mentoring instead of group interactions, we did not include this in the search for GPC programs. These search words were combined with the following search terms for specific populations. For diabetes, we included “gestational diabetes mellitus,” “GDM,” “type 2 diabetes,” “type 2 diabetes mellitus,” “type II diabetes,” “type II diabetes mellitus,” or “T2DM.” For pregnant women who smoked, we included “tobacco,” “cigarette,” “tobacco products,” or “addiction.” For women with opioid addiction, we included “opioid use disorder,” “opioid agonist treatment,” “opioid substitution treatment,” “naltrexone,” “medication assisted treatment,” “substance abuse,” “addiction,” “methadone,” or “buprenorphine.” For adolescents, we included “adolescent” or “teen.” For African Americans, we included “African American” or “black.” For low-income and homeless, we included “homeless,” “low-income,” or “poverty.” While not used as a search term, Medicaid-eligibility was used as a proxy for low-income in the selection of articles for that population. For overweight and obese women, we included “overweight,” “obese,” or “BMI.” For women with HIV/AIDS, we included “HIV,” “AIDS,” or “human immunodeficiency virus.” To further expand our search, we reviewed articles listed in the reference section for CenteringPregnancy® on Centering Health International’s website (www.centeringhealthcare.org) and searched the reference lists of articles found during our search. We excluded reports of GPC in unselected populations that were predominantly low-risk and did not report specifically on the groups above. All searches were completed between August 2016 and March 2017. Articles were included if they demonstrated results of implementation of GPC in the specific high-risk selected population, regardless of study type. We did not restrict study type. Identified studies were evaluated independently by both authors for risk of bias and outcomes. Any disagreements in assessments were resolved through consensus. RCTs were evaluated using the Cochrane Collaboration risk of bias assessment in the Cochrane Handbook and nonrandomized studies were assessed using the ROBINS-I tool [22, 23]. For reports that reported results of GPC groups without a control group comparison no formal evaluation of risk of bias was performed.

Second, to find current ongoing programs, we contacted Centering Health International, who provided us a list of current programs to their knowledge. We used Google’s search engine to identify additional programs. We then contacted leaders of identified programs requesting information regarding types of programs offered and any results or publications arising from those programs. A few leaders identified additional programs, with whom we also attempted to establish contact. All contact was by email or by phone.

We collected data from the identified studies and ongoing groups pertaining to the groups, programs, and outcomes. We collected study type (randomized trial or observational cohort), the specific selection criteria for the groups, and the outcomes reported, both maternal outcomes such as pregnancy complications, breastfeeding rates, and any satisfaction outcomes, and infant outcomes such as gestational age at birth, birth weight, and any adverse outcomes reported by the trial. Any outcome comparisons to control groups that had been obtained as part of program evaluation were collected from individual ongoing group coordinators identified, in addition to any variables described above. As many of these groups did not contain a control group, however, the descriptive statistics and outcomes are presented.

As the data sources and cohorts had tremendous heterogeneity, no meta-analysis or outcome combination was performed. Results are collated in descriptive tables. Within-trial comparisons are presented in the table.

No review protocol exists and the systematic review was not registered.

## Results and Discussion

### Search results

**Fig. 1:**
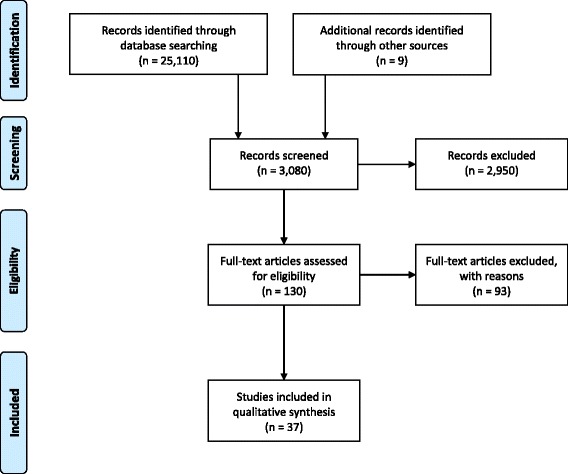
PRISMA 2009 flow diagram

The search yielded a total of 6537 articles on adolescents, 2454 articles on African American women, 2476 on low-income women, 1207 on overweight women, 880 on women with tobacco use, 960 on women with opioid use, 10,172 on women with diabetes, and 424 on women with HIV/AIDS. Titles and abstracts were reviewed to determine relevance. Studies were excluded from this review for a multitude of reasons, including not utilizing group prenatal care, the target population not being a selected high-risk group, and the article being written in a language other than English (Fig. 1). In the end, 37 reports were determined the most relevant and the descriptive results are presented below and in Additional file 1: Table S1. These included 8 reports of 4 RCTs, 23 reports of 21 nonrandomized studies, and 6 reports of outcomes from groups with no control group. Risk of bias assessments are presented in Table 1. Summaries of data for the individual groups are reported below.

**Table 1:** Risk of bias assessment summary of studies

Study	Random sequence generation (selection bias)	Allocation concealment (selection bias)	Blinding of participants and personnel (performance bias)	Blinding of outcomes assessors (performance bias)	Incomplete outcome data (attrition bias)	Selective reporting (reporting bias)	Other bias
Randomized trials							
Ford 2002 [32]	?	?	?	?	+	?	+
Ickovics 2007 [5]	+	+	–	+	+	+	?
Ickovics 2016 [8]	+	+	–	?	+	+	+
Klerman 2001 [29]	?	+	–	?	?	+	+
Nonrandomized studies	Bias due to confounding	Participant selection bias	Intervention classification bias	Deviation from intended intervention bias	Incomplete data bias	Measurement of outcome bias	Reported result selection bias
Adams 2016 [31]	+	+	++	++	+	++	++
Bloom 2005 [35]	+	+	++	++	+	++	++
Chwah 2016 [48]	+	+	++	++	+	++	++
Gareau 2016 [41]	+	+	++	++	++	++	++
Grady 2004 [1]	+	+	+	+	+	+	++
Griswold 2012 [37]	+	+	+	–	+	+	+
Heberlein 2016^a^ [42]	+	+	++	++	++	++	++
Heberlein 2016 [44]	+	+	++	++	++	++	++
Hieronymus 2016	+	+	–	++	++	++	++
Ickovics 2003	+	++	++	++	++	++	++
Jacobs 2016 [38]	+	+	++	+	++	++	+
Klima 2009 [16]	+	+	+	+	+	–	–
Kominiarek 2017 [49]	+	+	++	++	++	++	++
Mazzoni 2015 [24]	+	+	++	++	++	++	++
Nguyen 2014 [27]	+	n/a	+	?	?	?	?
Parikh 2016 [26]	+	+	++	++	++	++	++
Picklesimer 2012 [3]	+	+	++	++	++	++	++
Ramirez 2015	+	+	+	?	?	?	?
Schellinger 2016 [25]	+	+	++	++	++	++	+
Tandon 2012 [4]	+	+	++	++	++	++	++
Tandon 2013 [44]	+	+	++	++	++	++	++
Trotman 2015 [10]	+	+	++	++	++	++	++
Zielinski 2014 [28]	+	+	+	++	++	++	++

Risk of bias assessed per Cochrane Handbook guidelines for RCTs [22] and using the ROBINS-I tool for nonrandomized studies [23]

For RCTs: + = low risk of bias,? = unclear risk of bias, − = high risk of bias

For nonrandomized studies: ++ = low risk of bias; + = moderate risk of bias; − = serious risk of bias; −− = critical risk of bias;? = no information or not enough information to make judgement

This table does not include reports without control groups or secondary analyses of the primary randomized trials above

^a^Heberlein study entitled, “Qualitative comparison of women’s perspectives on the functions and benefits of group and individual prenatal care”. Other Heberlein entry is for “Effects of group prenatal care on food insecurity during late pregnancy and early postpartum”

### Quality of evidence

Assessment of the risk of potential bias for individual studies is presented in Table 1. As noted in this summary and the meta-analysis by Carter, much of the evidence supporting GPC carries a high potential risk of bias [13]. The rigor of RCTs can help reduce the risk of bias and thus more RCTs in these high-risk populations are needed to determine if observational benefits translate to specific high-risk populations. It is encouraging that the only high-risk subgroup analysis done in the Carter meta-analysis of African-American women did show that high-level evidence demonstrated a reduction in preterm birth. This was in contrast to the findings overall [13]. Focusing on high-risk groups may be a means for GPC to have the most benefit in improving outcomes.

### Diabetes

Although few studies have analyzed the impact of GPC in women with diabetes, those that have report promising results. Two studies demonstrated that significantly fewer women with GDM required treatment with medication when exposed to GPC compared to controls [24, 25]. Furthermore, for those women requiring treatment with insulin, GPC individuals required less than half the dose compared to controls (*p* < 0.001; [24]). Both studies demonstrated increased rate of neonatal hypoglycemia for infants born to GPC women, but there was no difference in the need to treat the hypoglycemia. There were no differences in neonatal outcomes such as preterm birth or NICU admission rates nor were there differences in the rates of cesarean section. Similar to the results of other studies, women in GPC were significantly more likely to attend their postpartum visit and to have a postpartum GTT performed. While these results are promising, the studies were performed with mostly Hispanic women, limiting generalizability. Further studies are required to determine the benefit to other populations.

Research involving both GDM and pre-gestational DM has not shown a large effect of GPC on outcomes. Women in GPC did not have a significantly different HbA1c compared to controls, which implies similarly controlled blood glucose levels despite intervention [26]. This was the only study found to include patients with pre-gestational diabetes. However, a decision-analytic model describing costs and pregnancy outcomes of women with type II diabetes mellitus attending GPC compared to traditional antenatal care demonstrated improved outcomes and cost-effectiveness of GPC “even when cost to attend group prenatal care was up to $11,000/pregnancy more than individual group care” [27].

### Tobacco use

Tobacco cessation has not been commonly studied in the setting of GPC. Zielinski’s group found that more women who selected GPC quit smoking at pregnancy diagnosis. In addition, they found that women who had continued to smoke after finding out they were pregnant were 5 times more likely to quit later in pregnancy if they were engaged in GPC (*p* < 0.001; [28]. While not significant, another study found a trend toward increased smoking cessation in women attending GPC compared with controls (50.0% vs 31.4%, *p* = 0.09; [29]. This may be due to provision of a social network to support them in smoking cessation that women may not have outside of GPC as social support is positively associated with smoking cessation (OR = 1.06, 95% CI (1.02–1.10), *p* < 0.01; [30]. Although smoking cessation is reportedly higher in GPC, this has not yet been found in association with improved neonatal or maternal health outcomes [28–30].

### Opioid addiction

Only one study of GPC for women with opioid addiction was found in the literature. In this study, no differences were found in preterm delivery <37 weeks, APGAR score at 5 min, or NICU admission [31]. Participants in GPC were less likely to be admitted to the emergency room triage during pregnancy (*p* = 0.02). They also attended more total visits than those in traditional prenatal care (*p* < 0.001). This study was limited by small sample size, differences in rates of multiple drugs used at the same time between groups, and had limited generalizability due to nearly all white participants.

### Adolescents

The reported effect of GPC in adolescent populations on neonatal outcomes has been inconsistent. One RCT showed a trend toward a decreased risk of low birth weight infants (6.6% GPC vs 12.5% control; *p* = 0.08; [32] while another showed no difference except in the subgroup that attended at least half of GPC visits (5.2% GPC vs 10.7% Control; *p* < 0.05; [8]). Inconsistency in outcomes is similarly found with preterm birth. A retrospective cohort study found no difference between groups [10], while an RCT found decreased preterm birth only in the subgroup that attended at least half of GPC visits (4.1% GPC vs 12.0% Control; p < 0.05; [8]). Observational studies showed similarly inconsistent results. NICU admission rates have not been found to be significantly different between GPC and traditional prenatal care [8, 10].

Participation in GPC did, however, result in adolescents’ increased compliance with appointments and adherence to pregnancy recommendations. Those enrolled in GPC were less likely to miss prenatal appointments [1, 8, 10, 32]. Attendance at postpartum visits was less often studied, and results were inconclusive [1, 10]. Adolescents in GPC were significantly more likely to adhere to IOM gestational weight gain guidelines, including retaining <10 lbs. postpartum [10, 33]. Significant benefits were seen even when controlling for baseline obesity (*p* < 0.01; [33]). Additionally, GPC was found to be associated with decreased depressive symptoms in adolescents. “Probable depression” diagnoses decreased by 31% for adolescents in GPC vs 15% for controls (*p* = 0.002, [34]).

Although only measured in one study, uptake of Long-Acting, Reversible Contraception (LARC) or long acting injectable medroxyprogesterone acetate use by the postpartum visit was found to be significantly increased in adolescents who participated in GPC [10]. One study only found a decrease in those attending at least half of the prenatal care sessions (*p* < 0.05; [8]). Effects of GPC on rapid repeat pregnancy are inconclusive [8, 10, 32, 35].

Other outcomes were also affected by GPC. GPC significantly increased rates of breastfeeding at discharge in adolescents [1, 10]. Pregnancy knowledge scores were significantly increased for GPC participants [33, 35]. GPC was associated with higher satisfaction scores than those for adolescents engaged in traditional prenatal care [1, 36]. These outcomes are consistent with previous studies in low-risk populations.

Of note, educational outcomes were found to be improved for adolescents participating in GPC. In a study of GPC offered in a high school for pregnant and parenting teens, 92% reported that the program encouraged them to attend school [37]. This translated to a 14.2% increased attendance rate compared with the overall student body the previous year [37]. Furthermore, an RCT found that adolescents participating in GPC completed 0.5 years of schooling more than women engaged in traditional antenatal care at one year postpartum (*p* < 0.01; [32]).

### African Americans

Multiple reports of unselected women using group prenatal care have significant numbers of racial and ethnic minority participants, including African Americans. When the analysis was limited to the two high-quality studies, the pooled rate of preterm birth for African Americans was 8.0% compared with 11.1% (pooled RR 0.55, 95% CI 0.34–0.88). This reduction was not seen for other racial or ethnic groups [13]. Several studies contained cohorts that were made up primarily of African Americans, but often not 100%. Ford’s trial in Detroit had a cohort which was 94% African American but did not show significant benefits in that group in preterm birth reduction [32]. Grady’s observational study which contained mostly African American women demonstrated lower rates of low birth weight and preterm birth but had uncharacteristically high rates of the outcomes in the control groups; preterm birth rate was 25% in the control women [1]. Another retrospective review did show lower preterm birth rates in a group of African American women receiving group care, but the study had a high risk of bias [38]. Some other studies that had majority populations of African Americans did not show reduced preterm birth rates but did show that group care improved breastfeeding rates and satisfaction with care [10, 16, 29].

Two studies used focus group methodology to evaluate group care in African American and low income women [16, 39]. These reports identified that group care can improve satisfaction and suggested that factors that may help improve outcomes in group care settings center around increasing social support systems and groups for parenting and being more aware of the available programs in place to provide resources [39].

Racial disparities in preterm birth and other adverse outcomes are striking in the United States and elsewhere [21, 40]. The published evidence, particularly that of high quality, support that group prenatal care may hold specific benefits for African American women, particularly regarding reduction in preterm birth, improved breastfeeding initiation, and satisfaction with care. Furthermore, the recent meta-analysis of group prenatal care highlighted that the benefits of group care are likely to be seen most in African American women [13]. The findings of this report further support this conjecture. However, more high-quality studies that limit potential biases and intentionally select cohorts of African American women are needed to confirm the benefit of GPC for this population.

### Low-income women

Many of the studies discussed above included a large number of women in low-income settings. Thus, the benefits seen in studies such as Ickovics, Griswold, Klerman, Picklesimer, and Trotman (which all had a majority of low income women) are likely generalizable to women in low income settings [3, 5, 10, 29, 33]. In addition to the studies described for other high-risk groups above, the RCT by Ickovics of low-income women showed a 33% decreased risk of preterm birth overall that was increased to a 41% decreased risk when analyzing results for African Americans alone [5]. No difference was found in other neonatal outcomes including low birth weight infants or NICU admission. Gareau also performed a 5 year retrospective review of Medicaid eligible pregnant women in group care and found a 36% reduction in preterm birth rates in South Carolina [41]. This translated into reductions in low birthweight and NICU admissions. This led to the group calculating a large return on investment in group prenatal care.

Several secondary outcomes were found to be improved. Ickovics’ group found acts of unprotected sex were decreased for those in GPC at 12 months postpartum (*p* = 0.05; [42]) while Kershaw’s group found decreased repeat pregnancy at 6 months postpartum (*p* = 0.02), increased communication with partners about safe sexual activity in the third trimester and 12 months postpartum (at 6 months *p* = 0.001, at 12 months *p* = 0.03), and increased condom use postpartum (at 6 months *p* = 0.07, at 12 months *p* = 0.007; [43]). Another study by Ickovics demonstrated an increased rate of breastfeeding at discharge (*p* < 0.001), increased knowledge scores (p < 0.001), and higher satisfaction with care than in a traditional setting (p < 0.001; [5]).

Other studies in low-income women that focused on psychosocial/societal outcomes demonstrate that group care can improve rates of food security compared to traditional care, particularly for women with food insecurity at the beginning of pregnancy [44]. While the Heberlein group did not find reduction in stressors for the pregnant women in group care, Novick et al. found that group care for low-income minorities reduced stressors by limiting wait times in clinic, allowing children to attend group sessions, extending time to learn, and by normalizing many of the stressors and anxieties of pregnancy in a low-income setting [45]. Additionally, Ickovick’s study found that women in GPC in the top tertile of stress early in pregnancy had decreased stress (*p* = 0.005), decreased social conflict (*p* = 0.008), and increased self-esteem (*p* = 0.009) in the third trimester [42], though only decreased social conflict remained significant post-partum (*p* = 0.004; [42]). Finally, Tandon’s study of low-income immigrants found that participation in group care increased the number of prenatal and postpartum visits attended and was associated with higher rates of establishing a medical home for their infants [46]. These outcomes were in addition to a lower rate of preterm birth (5% vs 13%, *p* = 0.04). While many of these studies are observational and have a high risk of bias, the consistency in improvements in these types of outcomes for low-income women is encouraging.

One study utilized semi-structured interviews with Medicaid-eligible women to analyze women’s perspectives on the benefits of GPC. Through comparisons of interview transcripts from women in GPC and traditional prenatal care, they found that the extra time allowed by GPC helped develop strong relationships with providers and other group members, providing social support [47]. Women in GPC reported greater benefits in stress reduction, confidence, knowledge, and motivation which was facilitated by the additional time provided, open question-and-answer time for the group, and the opportunity to learn from other women [47]. They also felt more prepared for labor, birth, newborn care, and breastfeeding [47].

### Overweight individuals

Few studies have been published that analyzed the effect of GPC on weight. One study was found that focused specifically on women with BMI > 30 and weight-related outcomes, but it was limited in its analysis of weight changes by incomplete data sets [48]. The study did find that those in GPC were more likely to receive nutritional, exercise, weight gain advice as well as regular weighing (*p* < 0.001; [48]). Maternal and neonatal outcomes were not significantly different except for assisted vaginal delivery (*p* = 0.016).

Another study focused on the association of GPC and gestational weight gain among Medicaid-eligible women. It showed associations between GPC and higher gestational weight gain as well as a higher proportion of women exceeding the Institute of Medicine’s weight gain recommendations for pregnancy [49]. Overall, those in GPC had a 30% greater risk of exceeding weight gain goals. This was concentrated among normal weight and overweight women with a 28% and a 94% increased risk respectively [49]. No difference was found between the two groups for underweight or obese women. Notably, this study was limited by significant differences in age, parity, SES, and high-risk medical conditions between GPC and control groups. Furthermore, the increased mean weight gain of 2 lbs. in normal weight women and 4 lbs. in overweight women has limited clinical significance.

A third study of weight gain patterns among pregnant women on Medicaid reported an average weight gain of 26 lbs. (range 2–65 lbs) by delivery and an average weight loss of 19 lbs. at the postpartum visit (range 2–24 lbs) among women in CenteringPregnancy® [50]. The study was limited by a small sample size (*n* = 74) and lack of a control group.

Two studies performed in adolescent populations used weight as a secondary outcome. Trotman found that those in GPC were more likely to meet the Institute of Medicine’s guidelines for gestational weight gain in a mostly African American population (*p* = 0.02; [10]). Magriples found a significant difference in weight trajectories between Latina and African American adolescents in GPC compared with traditional care (*p* < 0.001; [34]). Individuals in GPC gained less weight during pregnancy and were more likely to meet guidelines for retaining <10 lbs. postpartum [34]. These differences persisted when stratified by obesity status (*p* < 0.01). Interestingly, this study highlighted the association between depression and prenatal distress and weight gain. They found that adolescents in GPC had similar weight trajectories regardless of depressive symptoms or prenatal distress while adolescents in control groups with moderate and high baseline levels had more weight gain during pregnancy and less weight loss postpartum (*p* < 0.0001; [34]). While these numbers are impressive, it is important to note that 20% of the original sample was excluded from analysis due to incomplete data, increasing the risk of overestimating the significance of these findings.

### Women with HIV/AIDS

We did not find published reports specifically reporting on use of GPC for pregnant women with HIV or AIDS. There are currently ongoing groups for women with these conditions described below.

### Current high-risk GPC programs

Several sites are currently recruiting specific groups of high-risk women to GPC programs. Adolescent groups are currently running in Ohio by the Adena Health System, which serves Appalachia. An adolescent prenatal group care program has also recently been initiated by Indiana University School of Medicine (IUSM) in Indianapolis, IN. Groups for women with diabetes, either gestational or pre-gestational, are being offered by the Adena, SUNY in upstate New York, and IUSM. Groups for women with opiate addiction are offered by the Adena, Summa Health in Akron OH, IUSM, the University of Kentucky, and the Health Share of Oregon. These programs will soon be offered by SUNY as well. Groups for women with a history of preterm birth are being offered by Adena, as are groups for women with heavy tobacco and caffeine use. Groups for women with HIV/AIDS are offered by Harris Health Northwest Health Center in Houston, TX and at the University of Miami in Florida. Groups for Nepali refugee women are offered by Summa Health in addition to groups for African American women. Groups for homeless women are currently being offered by the Homeless Prenatal Program in San Francisco, CA. As of the submission of this article, outcomes were not yet available for these high-risk programs. Additionally, there are likely many other centers offering group prenatal care to specific high-risk groups that we did not discover.

## Conclusions

Group prenatal care may provide advantages over the traditional model of care. There are multiple branded models of group care, each with varying degrees of evidence supporting the benefits. While the recent meta-analysis demonstrated that in general, GPC did not lead to reduced rates of preterm birth, NICU admission, and breastfeeding, the subgroup of African-American women was different. By teasing out the different high-risk groups, it is possible that the benefits of group care may become more evident. In this way GPC could become a targeted intervention to improve outcomes.

As highlighted above, many authors have reported outcomes with various high-risk groups engaging in GPC. The groups, unfortunately, often overlap in their risk categories. Additionally, the reports are often observational in nature and subject to selection and reporting biases. However, there is some consistency in that several high-risk groups may have benefits from engaging in group prenatal care- both in obstetric outcomes such as preterm birth and breastfeeding, and also in psychosocial outcomes such as improved satisfaction, reduced stress, understanding resources, and engagement in their own and their family’s health care.

The purpose of this review was to summarize the current state of literature reporting on group prenatal care for high-risk pregnant women. There is a paucity of high-quality trial evidence in these women. Given the cost savings noted in several studies, more trials of group care in high-risk women should be undertaken to truly determine if group care improves outcomes and costs of pregnancy-related care for high-risk women. While RCTs would be ideal, cohort studies that are more intentional in their limitation of bias through measures such as inclusion of control groups and collecting baseline data to be used to correct for potential confounding variables are most practical in furthering knowledge on this topic. Additionally, these studies need to follow the families longer term to report on engagement in health behaviors after the immediate postpartum period and the health of the children from these pregnancies. A comprehensive analysis of the components of group prenatal care that are the keys to improved outcomes should also be explored. Group prenatal care advertises improved pregnancy outcomes and these outcomes should be demonstrated for high-risk pregnant women as well.

## Additional file

Additional file 1: Summary of Articles on Group Prenatal Care in High-Risk Populations. (DOCX 26 kb)

## Abbreviations

AIDS: Acquired immune deficiency syndrome
DM: Diabetes mellitus
GDM: Gestational diabetes mellitus
GPC: Group prenatal care
GTT: Glucose tolerance test
HIV: Human immunodeficiency virus
LARC: Long-acting reversible contraception
NICU: Neonatal intensive care unit
RCT: Randomized controlled trial
