# Supplementary material for: A systematic overview of the literature regarding group prenatal care for high-risk pregnant women
Source: BMC Pregnancy Childbirth. 2017 Sep 29;17:329. doi: 10.1186/s12884-017-1522-2 (PMC5622470; doi:10.1186/s12884-017-1522-2)
Supplement: Additional file 1: — Summary of Articles on Group Prenatal Care in High-Risk Populations. (DOCX 26 kb) [file 12884_2017_1522_MOESM1_ESM.docx]

| Table 1: Summary of Articles on Group Prenatal Care in High-Risk Populations. | | |  |
| --- | --- | --- | --- |
| Author(s)/Publication Year | Article Title | Population | Findings |
| Hieronymus, L., Combs, L., Coleman, E. Ashford, K. & Wiggins, A. (2016) | Evaluation of an education intervention in Hispanic women at risk for gestational diabetes mellitus | Hispanic women at high risk of GDM | 86% rated topics "very helpful", 93% liked prenatal group format, felt comfortable in group setting, felt prepared for labor, birth, and parenting |
| Mazzoni, S. E., Hill, P. K., Webster, K. W., Heinrichs, G. A. & Hoffman, M. C. (2015) | Group prenatal care for women with gestational diabetes | Women with diagnosed GDM; majority Hispanic | Less likely to progress to A2 GDM (OR 0.15, CI 0.07-0.30), insulin required at less than half the rate in GPC compared with controls (26% vs 63%; p < 0.001), more likely to attend postpartum visit (92% vs 66%, p = 0.002) and to receive recommended diabetes screening postpartum (OR 3.9) |
| Schellinger, M. M., Abernathy, M. P. & Amerman, B. et al. (2016) | Improved outcomes for Hispanic women with gestational diabetes using the Centering Pregnancy group prenatal care model | Hispanic women with diagnosed GDM | Fewer women needed drug therapy (p = 0.009), more likely to complete postpartum glucose tolerance testing (83.6% vs 60.7%, p < 0.001), higher rate breastfeeding at hospital discharge (91.0 vs 69.4%, p < 0.001), less likely to undergo inductions of labor (34.5 vs. 46.2 %; p = 0.014) |
| Nguyen, N., Allen, A. & Gorman, M. et al. (2014) | Group prenatal care for women with pre-gestational type II diabetes mellitus: a cost-effectiveness analysis | Women with pre-gestational type 2 diabetes mellitus | Fewer cases of preterm birth, intrauterine fetal demise, preeclampsia. GPC is cost-effective. |
| Parikh, L. I., Jelin, A. C. & Iqbal, S. N. et al. (2016) | Glycemic control, compliance, and satisfaction for diabetic gravidas in centering group care | Women with pre-gestational diabetes and GDM | Lower mean fasting blood sugar (91.0 vs 105.5, p = 0.017). No difference in change in fasting blood sugar over time. Better patient satisfaction scores |
| Masho, S. W., Do, E. & Adekoya, S. (2014) | Social support and smoking during pregnancy | Pregnant women during first trimester | Per unit increase in the total composite social support scale, there was a 6% increased odds of smoking during pregnancy. Suggest use of group prenatal care models to decrease smoking during pregnancy. |
| Zielinski, R., Stork, L., Deibel, M., Kothari, C. L. & Searing, K. (2014) | Improving infant and maternal health through CenteringPregnancy: a comparison of maternal health indicators and infant outcomes between women receiving group versus traditional prenatal care | Pregnant women | Increased rate of tobacco cessation (84% vs 24%, p < 0.000). More women who chose Centering quit smoking at diagnosis compared to standard care, but 50% of GPC women who had continued to smoke quit compared to 8% of control group (p < 0.000). |
| Adams, J., McCarroll, M., Frantz, K. et al. (2016) | Comparison of outcomes in maternal opioid medical support using Centering Pregnancy versus maternity care home | Opioid addicted pregnant women | Increased patient compliance with prenatal visits, including clinic visits and ultrasound visits (GPC 17.4 +/- 6.72 vs Control 8.6 (+/- 7.50); p < 0.001). Decreased visits to ER/OB triage (1.8 ± 2.03 visits versus control 3.2 ± 2.47 visits, p=0.020). No difference in preterm birth or NICU admission. |
| Ellison, T. (2010) | Adolescent group prenatal care: a pilot study evaluating patient satisfaction | Pregnant adolescent women, age 12-19 years | Satisfaction with group care rated 9.29/10; Report decreased anxiety as group shows them their concerns are common; Report being able to relate to other group members |
| Felder, J. N., Epel, E., Lewis, J. B. et al. (2017) | Depressive symptoms and gestational length among pregnant adolescents: cluster randomized control trial of CenteringPregnancy® Plus group prenatal care | Pregnant adolescent women, age 14-21 | Probable depression decreased by 31% (52 to 36%) for GPC; 15% (47 to 40%) for control. Significant PNC Type x Time interaction for depressive symptoms (F = 9.26, p = 0.002). Significant Type x Time interaction overall from baseline to 12-mo postpartum (p - 0.002) but not from baseline to 3rd trimester or baseline to 6-mo postpartum. |
| Ford, K., Weglicki, L., Kershaw, T. et al. (2002) | Effects of a prenatal care intervention for adolescent mothers on birth weight, repeat pregnancy, and educational outcomes at one year postpartum | Pregnant adolescent women, age 13-21 years | Decreased rate LBW infants (6.6% GPC vs 12.5% control, p = 0.08); Trend of decreased repeat pregnancy within next year (13.4% vs 15.9%, p = 0.52). GPC participants had completed 0.5 more years of education since the intake interview (p < 0.01) |
| Grady, M. A. & Bloom, K. C. (2004) | Pregnancy outcomes of adolescents enrolled in a CenteringPregnancy program | Pregnant adolescents, age 11-17 years | Lower no-show rate (19% vs 28%, p < 0.05), lower incidence of preterm births (10.5% vs 23.2%) and fewer LBW infants (8.87% vs 18.3%, p < 0.05). No difference in cesarean births. 87% had postpartum visit within 8 weeks after delivery. |
| Griswold, C. H., Nasso, J. T., Swider, S. et al. (2012) | The prenatal care at school program | Pregnant adolescents, age 14-19 years | Decreased absences from school; 92% stated the program encouraged them to attend school. Increased attendance at prenatal care classes. 42% increase in knowledge of prenatal care, labor/delivery |
| Ickovics, J. R., Earnshaw, V., Lewis, J. B. et al. (2016) | Cluster randomized controlled trial of group prenatal care: perinatal outcomes among adolescents in New York City health | Pregnant adolescents, age 14-21 years | GPC decreased risk of SGA infants; when they did, born at later gestational age. More group visits resulted in decreased risk SGA, preterm, and LBW infant, decreased # days in NICU, Decreased risk rapid repeat pregnancy, increased condom use (p = 0.03 to < 0001) |
| Magriples, U., Boynton, M. H., Kershaw, T. S. et al. (2015) | The impact of group prenatal care on pregnancy and postpartum weight trajectories | Pregnant adolescents, age 14-21 years | CP+ improved weight trajectories compared with controls (gained less weight during pregnancy and lost more weight postpartum, p < 0.0001). Effect sustained among obese women (p < 0.01). |
| Trotman, G., Chhatre, G., Darolia, R. et al. (2015) | The effect of Centering Pregnancy versus traditional prenatal care models on improved adolescent health behaviors in the perinatal period | Pregnant adolescents, age 11-21 years | More likely to comply with prenatal and postpartum visits, meet weight gain guidelines (p = 0.02), to breastfeed (p = 0.03), use LARC or DMPA. Less likely to have postpartum depression. |
| Bloom, K. C. (2005) | Use of the CenteringPregnancy program in a school-based clinic: a pilot study | Pregnant adolescents, age 14-18 | Increased knowledge scores (p < 0.05) but no difference in preterm birth or low birthweight infants |
| Tucker-Edmonds, B., Mogul, M. & Shea, J. A. (2015) | Understanding low-income African American women's expectations, preferences, and priorities in prenatal care | Low-income African American women | Top prenatal care attendance motivators: Friends/family and baby health. Barriers: insurance, transportation, ambivalence. Perfect system: continuity of care, personal connection, caring/respect from providers. |
| Jacobs, S. (2016) | Outcomes of CenteringPregnancy® in African-American Women | African American women | GPC went a longer number of weeks gestation (39.44 vs 38.66, p = 0.004); had fewer preterm births (p = 0.015) and no difference in rate of cesarean birth (p = 0.4255) |
| Klerman, L. V., Ramey, S. L. & Goldenberg, R. L et al. (2001) | A randomized trial of augmented prenatal care for multiple-risk, Medicaid-eligible African American women | Multiple risk, Medicaid-eligible African American women | No significant difference in neonatal or delivery outcomes. More smokers in augmented care quit smoking. Augmented care group rated care more helpful and knew more about risk conditions. |
| Cozianu, T., Kural, Y. & Leon, J. C. et al. (2016) | Centering Pregnancy impact on patterns of weight gain in an inner-city community teaching hospital | Pregnant women on Medicaid | Average weight gain by delivery was 26 lbs (range 2-65 lbs). Average weight loss at postpartum visit (ranged from 5-8 wks postpartum) was 19 lbs (range 2-24 lbs). |
| Gareau, S., Lopez-De Fede, A. & Loudermilk, B. L. et al. (2016) | Group prenatal care results in Medicaid savings with better outcomes: a propensity score analysis of CenteringPrengnancy participation in South Carolina | Pregnant women on Medicaid | 36% decreased risk premature birth (p < 0.05). 44% decreased risk LBW infant (p < 0.05). 28% decreased risk NICU stay (p < 0.05). Centering Pregnancy is cost-effective. |
| Heberlein, E. C., Picklesimer, A. H., Billings, D. L. et al. (2016) | Qualitative comparison of women's perspectives on the functions and benefits of group and individual prenatal care | Medicaid-eligible women | GPC allowed extra time that helped develop strong relationships with providers. GPC: greater benefits in stress reduction, confidence, knowledge, motivation, and informed decision making was facilitated by additional time, open Q&As as a group, and opportunity to learn from other women. GPC women also reported building relationships with other group members provided added support. They also felt more prepared for labor, birth, newborn care, and breastfeeding. |
| Heberlein, E. C., Frongillo, E. A., Picklesimer, A. H. & Covington-Kolb, S. (2016) | Effects of group prenatal care on food insecurity during late pregnancy and early postpartum | Racially diverse, low-income pregnant women | More likely to report food security late in pregnancy (p < 0.001) and postpartum (p = 0.049). Among those initially food-insecure, more likely to become food-secure in late pregnancy (p < 0.001) and postpartum (p = 0.052). |
| Ickovics, J. R., Reed, E., Magriples, U. et al. (2011) | Effects of group prenatal care on psychosocial risk in pregnancy: results from a randomized controlled trial | Low-income pregnant women age 14-25 | No difference based on intention to treat analysis. Those at top tertile of stress early in pregnancy assigned to CP+ had significantly better psychosocial outcomes (increased self-esteem, decreased stress, social conflict, and postpartum depression, p < 0.02). |
| Ickovics, J. R., Kershaw, T. S., Westdahl, C. et al. (2007) | Group prenatal care and perinatal outcomes: a randomized controlled trial | Low-income, predominantly African American and Hispanic women age 14-25 | GPC decreased risk preterm birth by 33% (OR = 0.67, p = 0.045). For AA analyzed alone, decreased risk by 41% (OR = 0.59, p = 0.02). No difference in neonatal outcomes. Increased knowledge and satisfaction with care (p < 0.001). |
| Ickovics, J. R., Kershaw, T. S. & Westdahl, C. et al. (2003) | Group prenatal care and preterm birth weight: results from a matched cohort study at public clinics | Low-income, predominantly African American and Hispanic women age 14-25 | Greater birth weight in infants not carried to term (P < 0.05). For preterm births, average gestational age 2 wks longer than individual prenatal care (p < 0.001). No difference in preterm delivery. |
| Kershaw, T. S., Magriples, U., Westdahl, C. et al. (2009) | Pregnancy as a window of opportunity for HIV prevention: effects of an HIV intervention delivered within prenatal care | Low-income, predominantly African American and Hispanic women age 14-25 | GPC decreased repeat pregnancy at 6 months postpartum, increased condom use postpartum, increased communication with partners about safe sexual activity in 3rd tri and 12 months postpartum. |
| Klima, C. Norr, K. Vonderheid, S. & Handler, A. (2009) | Introduction of CenteringPregnancy in a public health clinic | Medicaid-eligible women age 14-25. | Higher satisfaction (p < 0.05). Increased number prenatal visits. Increased weight gain during pregnancy (p = 0.05). Increased initiation of breast feeding (p = 0.05). No difference in neonatal outcomes. |
| Kominiarek, M. A., Crockett, A., Covington-Kolb, S., Simon, M., & Grobman, W. A. (2017) | Association of group prenatal care with gestational weight gain | Medicaid-eligible women | CenteringPregnancy associated with higher gestational weight gain and a higher proportion exceeding the IOM's gestational weight gain recommendations. This was concentrated among normal weight and overweight women. No difference between the two groups in underweight and obese women. |
| Moleti, C. A. (2015) | Centering Pregnancy Implementation and its Effect on Preterm Birth and Low Birthweight | Low-income women, mostly ethnic minority | GPC decreased preterm births compared to institutional average. Increased initiation of breastfeeding compared to institutional average. |
| Novick, G., Sadler, L. S., Knafl, K. A., Groce, N. E. & Kennedy, H. P. (2012) | The intersection of everyday life and group prenatal care for women in two urban clinics | Low-income, African American and Hispanic women | Extended time enhanced learning. Group had similar problems, so helped normalize concerns, reduce anxiety, decrease feelings of isolation and levels of stress. Report strengthened relationships with partners and within the community. |
| Picklesimer, A. H., Billings, D., Hale, N., Blackhurst, D. & Covington-Kolb, S. (2012) | The effect of CenteringPregnancy group prenatal care on preterm birth in a low-income population | Low-income women | Decreased rates preterm delivery (7.9 vs 12.7%, p = 0.01). Decreased rates delivery at < 32 weeks gestation (1.3 vs 3.1%, p = 0.03). GPC was protective for preterm delivery (OR = 0.53). No difference in rates of LBW infants < 2500 g or NICU admission. |
| Ramirez, S. (2015) | Advocating for the undocumented, pregnant Latino women: utilization and outcomes of Centering Pregnancy prenatal care in an urban, community setting | Undocumented, pregnant Latina women | Increased average gestational age at delivery, decreased LBW infant, decreased Cesarean delivery. Increased number exclusively breastfeeding infants at hospital discharge. |
| Tandon, S. D., Cluxton-Keller, F., Colon, L., Vega, P. & Alonso, Alina (2013) | Improved adequacy of prenatal care and healthcare utilization among low-income Latinas receiving group prenatal care | Low-income Latina women | Higher rate of satisfaction with prenatal care (p < 0.001). Higher percentage receiving adequate prenatal care (p < 0.001). More likely to establish medical home for child (p < 0.01). More likely to have postpartum checkup within 6 wks of delivery (p = 0.04). |
| Tandon, S. D., Colon, L., Vega, P., Murphy, J. & Alonso, A. (2012) | Birth outcomes associated with receipt of group prenatal care among low-income Hispanic women | Low-income Hispanic or Mayan women | Decreased number preterm births (p = 0.04). No difference in LBW infants. |
| Chwah, S. R., Reilly, A., Hall, B., O'Sullivan, A. J. & Henry, A. (2016) | Engagement with and outcomes of a midwifery-led intervention group for pregnant women of high body mass index | Women with BMI > 30 | Better implementation of high BMI guidelines (nutritional/weight gain advice (p < 0.001), regular weighing (p < 0.001), lactation consultant referrals (p = 0.02), third trimester anesthetic review and ultrasound (p = 0.04)). Increased breastfeeding initiation (p = 0.001). No difference in rate of Cesarean or birth weight. |
|  |  |  |  |
|  | Key: |  |  |
|  | Diabetes = yellow |  |  |
|  | Tobacco = light green |  |  |
|  | Opioid = dark green |  |  |
|  | Adolescent = orange |  |  |
|  | African American = light blue |  |  |
|  | Low Income = gray |  |  |
|  | Overweight = dark blue |  |  |
